# Morphological and Molecular Identification of Obligatory Myiasis-Causing Species in Wild Cervids in Croatia

**DOI:** 10.3390/ani15020208

**Published:** 2025-01-14

**Authors:** Ema Gagović, Daria Jurković Žilić, Krunoslav Pintur, Adnan Hodžić, Šimun Naletilić, Relja Beck

**Affiliations:** 1 Department of Bacteriology and Parasitology, Croatian Veterinary Institute, 10000 Zagreb, Croatia; gagovic@veinst.hr (E.G.); jurkovic@veinst.hr (D.J.Ž.); 2 Department of Wildlife Management and Nature Conservation, Karlovac University of Applied Sciences, 47000 Karlovac, Croatia; krunoslav.pintur@vuka.hr; 3 Centre for Microbiology and Environmental Systems Science (CMESS), Department of Microbiology and Ecosystem Science, University of Vienna, 1010 Vienna, Austria; adnan.hodzic@univie.ac.at; 4 Department of Pathology, Croatian Veterinary Institute, 10000 Zagreb, Croatia; naletilic@veinst.hr

**Keywords:** *Hypoderma diana*, *Cephenemyia simulator*, *Pharyngomyia picta*, *Capreolus capreolus*, *Cervus elaphus*, detection

## Abstract

The infestation of ruminants by fly larvae of the Oestridae family can lead to nasopharyngeal or subcutaneous myiasis, which can harm animal welfare and productivity on sheep and cattle farms. The infestation of wild ruminants allows them to serve as reservoirs for subsequent transfer to domestic animals. The fly species most commonly responsible for myiasis have been well studied in many parts of the world, but less so in southeastern Europe. Here, we identified three botfly species among 197 subcutaneous larvae and 138 nasopharyngeal larvae from 36 wild roe deer (*Capreolus capreolus*) and 9 wild red deer (*Cervus elaphus*) at 30 locations across Croatia. Instars were stored in 75% ethanol and their species and developmental stage were identified based on morphological and morphometric keys. Subcutaneous larvae in both cervid species were identified as *Hypoderma diana*, while nasopharyngeal larvae were identified as *Cephenemyia simulator* in roe deer and *Pharyngomyia picta* in red deer. Partial sequencing of the gene-encoding mitochondrial cytochrome oxidase subunit I confirmed the taxonomic classification of *C. stimulator* and *P. picta* but was unable to confirm that of *H. diana* for lack of previously published sequences. This study appears to be the first to combine the morphological and molecular characterization of myiasis-causing botflies in wild cervids in southeastern Europe.

## Simple Summary

The larvae of the Oestridae family parasitize the tissues, body cavities and gastrointestinal tract of mammalian hosts and thereby cause obligatory subcutaneous, nasopharyngeal and gastrointestinal myiasis, affecting wild and domestic mammals worldwide. The study aimed to present initial morphological and molecular findings regarding nasopharyngeal and subcutaneous myiasis in red deer and roe deer populations in Croatia. Larval specimens were obtained from 45 wild cervids during the timeframe spanning from 2015 to 2024. Subcutaneous larvae in both cervid species were identified as *Hypoderma diana*, while nasopharyngeal larvae were identified as *Cephenemyia simulator* in roe deer and *Pharyngomyia picta* in red deer. Our results provide a reference for similar investigations elsewhere in southeastern Europe, which are important for obtaining a complete understanding of botfly distribution throughout Europe as climate conditions become increasingly favorable to their growth.

## 1. Introduction

While adult flies of the Oestridae family live freely, their larvae require a vertebrate host to complete their life cycle [1]. The larvae parasitize the tissues, body cavities and gastrointestinal tract of mammalian hosts and thereby cause obligatory subcutaneous, nasopharyngeal and gastrointestinal myiasis, affecting wild and domestic herds of ruminants and equids worldwide [2]. The infestation of domestic animals can immunosuppress them, increasing risk of secondary infections that reduce animal well-being, hide quality and milk production [3,4]. The infestation of wild cervids has been linked to weight loss and the severe inflammation of the nasal mucosa [5,6].

The diverse species of the Oestridae family have adapted to a wide range of ruminant hosts. In western and southeastern Europe, species of the genus *Hypoderma* (subfamily Hypodermatinae) are the most frequent cases of myiasis, including *H. bovis* (Linnaeus, 1758) and *H. lineatum* (Villers, 1789) in cattle (*Bos taurus*, Linnaeus, 1758); *H. diana* (Brauer, 1858) and *H. actaeon* (Brauer, 1858) in roe deer (*Capreolus capreolus*, Linnaeus, 1758) and red deer (*Cervus elaphus*, Linnaeus, 1758); and *H. tarandi* (Linnaeus, 1758) in reindeer (*Rangifer tarandus*, Linnaeus, 1758) [7]. Nasopharyngeal botflies of the subfamily Oestrinae also show different host preferences depending on fly species [8], with *Cephenemyia auribarbis* (Meigen, 1824) infecting primarily red deer; *Cephenemyia stimulator* (Clark, 1815) infecting roe deer; *Cephenemyia ulrichii* (Brauer, 1862) infecting moose (*Alces alces*, Linnaeus, 1758); *Oestrus ovis* (Linnaeus, 1758) infecting domestic sheep (*Ovis aries*, Linnaeus, 1758), goats (*Capra hircus*, Linnaeus, 1758), European mouflon (*Ovis gmelini musimon*, Pallas, 1811) and alpine ibex (*Capra ibex*, Linnaeus, 1758); and *Pharyngomyia picta* (Meigen, 1824) infecting red deer, sika deer (*Cervus nippon*, Linnaeus, 1758), fallow deer (*Dama dama*, Linnaeus, 1758), Iberian red deer (*Cervus elaphus hispanicus*, Hilzheimer, 1909) and roe deer [9,10]. In addition to mammals, certain species of oestrid flies, including *O. ovis*, *H. tarandi*, and *C. ulrichii*, have the potential to infect humans [11,12]. 

Much less is known about the botfly species responsible for myiasis among domestic and wild ruminants in southeastern Europe. Croatia is a good area to examine this question because it contains continental and Mediterranean regions suitable for various oestrid fly species and their wild cervid hosts, particularly roe deer and red deer, which are the most common wild cervid hosts in other parts of Europe [13,14,15]. Studies based solely on morphology have reported *Cephenemyia stimulator*, *C. ulrichii*, and *P. picta* in roe deer and red deer in Croatia [16,17], and one such study reported a 12% prevalence of *C. stimulator* in roe deer in one part of the country [18]. Those studies did not cover most of the country, nor did they validate their species identification using molecular techniques. The present study therefore undertook the morphological and molecular characterization of botflies linked to subcutaneous and nasopharyngeal myiasis in roe deer and red deer across Croatia.

## 2. Materials and Methods

Larvae were sampled from wild cervids that had been killed within legally sanctioned game management plans or that had been found dead and sent to the Pathology Laboratory at the Croatian Veterinary Institute (Zagreb, Croatia). A total of 36 roe deer and 9 red deer were sampled between 2015 and 2024. 

Larvae were analyzed for developmental stage, and third-instar larvae were analyzed for species based on morphological criteria that included the shape and arrangement of the antennal lobes, as well as the presence or absence of the cephalic spines at the anterior part; the shape of the peritremes at the posterior ends; and the location, orientation, distribution pattern and shape of cuticular spines [1,7,8,19]. Morphology was examined under a Stereo Discovery 20 microscope (Zeiss, Jena, Germany) and an Imager M.2 microscope (Zeiss) equipped with Axiovision and ZEN2 Pro software (blue edition, version number 3.5.093.00010 (Zeiss). 

After preservation in absolute ethanol at −21 °C, larvae were analyzed molecularly to determine the species. From each animal, 1–8 larvae in the second- or third-instar stages were analyzed. A piece of tissue weighing ~20 mg was cut from each larva and placed into a 2 mL microcentrifuge tube, and then DNA was extracted using the King Fisher and Mag Max Core extraction kit (Thermo Fisher Scientific, Waltham, MA, USA) following the manufacturer’s instructions. The DNA was eluted in 100 μL of elution buffer, and a region of the gene-encoding mitochondrial cytochrome oxidase subunit I was amplified using primers LCO1490 and HCO2198 [20] in 20 μL PCR reactions containing 1 μL of DNA template, 0.4 μL of each primer (10 pmol/μL), 10 μL of G2 GOTaq Mastermix (Promega, Madison, WI, USA) and 8.2 μL of DNase/RNase-free distilled water (Promega). During all amplifications, positive and negative controls were run in parallel. Amplified DNA was analyzed using the QIAxcel system of capillary electrophoresis and DNA Fast Analysis (Qiagen, Hilden, Germany), and amplicon size was estimated using DNA QX Alignment Markers 15 bp/3 kb and QX DNA Size Markers 50–3000 bp (Qiagen). 

The amplicon of the expected size was purified using the EXOSAP-it^®^ kit (Thermo Fisher Scientific, Waltham, MA, USA) according to the manufacturer’s instructions, then sequenced in both directions using the same primer pair by the firm Macrogen (Amsterdam, The Netherlands). Sequences were assembled using SeqMan Pro 17 and edited using SeqBuilder Pro 17 with the Lasergene Molecular Biology software 17.6 suite (DNASTAR, Madison, WI, USA), then input into the Basic Local Alignment Search Tool (https://blast.ncbi.nlm.nih.gov) to identify relevant sequences in GenBank^®^ (accessed on 18 December 2024).

The sequences from the present study were aligned to sequences in the database using the ClustalW algorithm with default parameters, as implemented in BioEdit 7.2.5 [20]. Aligned sequences were manually trimmed to cover 591 nucleotides. 

Phylogenies were reconstructed using maximum likelihood analysis (as implemented in MEGA 7.0 [21]. Based on the corrected Akaike information criterion, the optimal nucleotide substitution model during reconstruction was found to be the general time-reversible model with gamma-distributed rate variation and a proportion of invariant sites (GTR + G + I). Phylogenetic trees were generated using the heuristic nearest-neighbor-interchange approach and 1000 bootstrap replicates to assess node support.

## 3. Results

**Figure 1:**
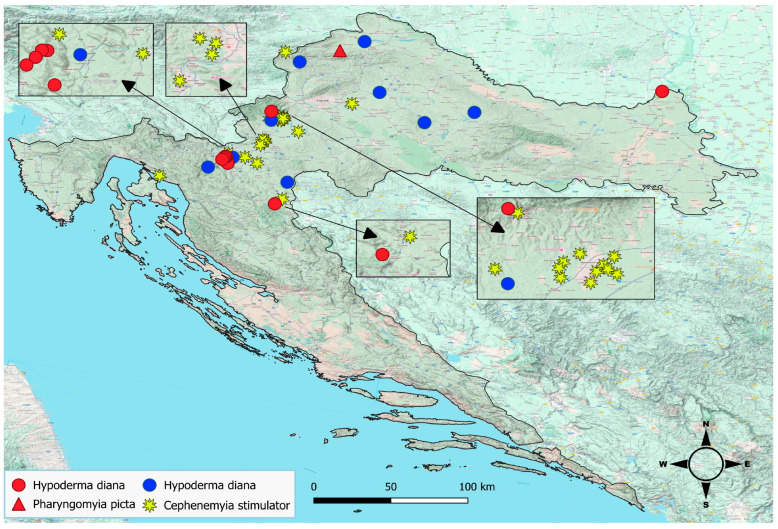
Locations of sampled roe deer (blue and yellow symbols) and red deer (red symbols) in Croatia, annotated according to the botfly species subsequently identified. Insets show zoomed regions of the map.

**Figure 2:**
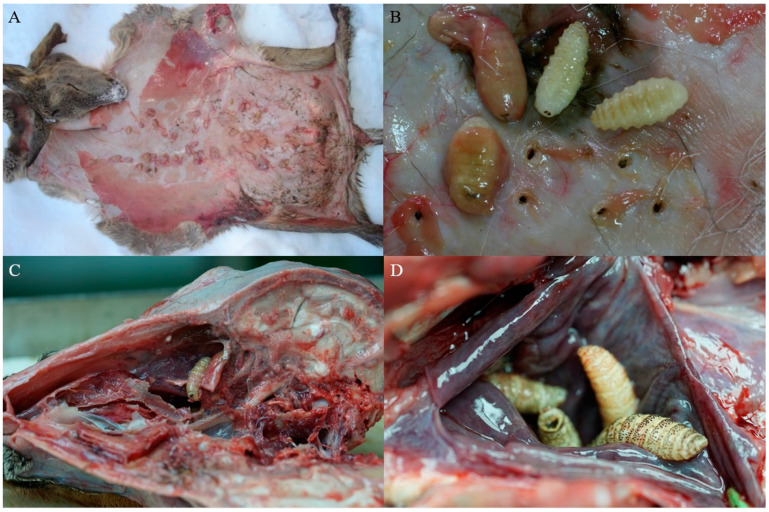
Examples of macroscopic findings in a roe deer. (**A**) Subcutaneous warbles. (**B**) Larvae of *Hypoderma* sp. extracted from subcutaneous warbles. (**C**,**D**) Larvae of *Cephenemyia* sp. in the pharyngeal pouch.

Fly larvae were sampled from wild cervids throughout the country that were brought to the Pathology Laboratory at the Croatian Veterinary Institute. One roe deer came from the northern coastal region of Croatia, while the others came from 19 continental locations and 10 mountainous locations (Figure 1). Larvae were found in the nasopharynx of 27 roe deer (125 larvae) and one red deer (13 larvae), while subcutaneous larvae were retrieved from nine roe deer (138 larvae) and eight red deer (59 larvae) (Figure 2). Details about the larvae and their hosts are provided in Table S1 in the Supplementary Materials.

**Figure 3:**
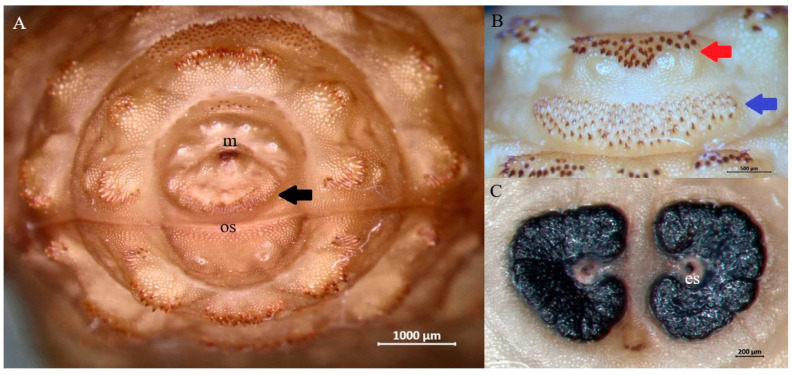
Representative third-instar larva of *Hypoderma diana*. (**A**) Anterior end showing the mouth (m), opercular suture (os) and band of spines between mouth and opercular suture (black arrow) on the cephalic segment. (**B**) Ventral spines on the first thoracic segment showing posteriorly directed spines with narrow bases and rounded tips (red arrow) and anteriorly directed spines with narrow bases and sharp tips (blue arrow). (**C**) Flat, C-shaped spiracular plates incompletely surrounding an ecdysal scar (es).

**Figure 4:**
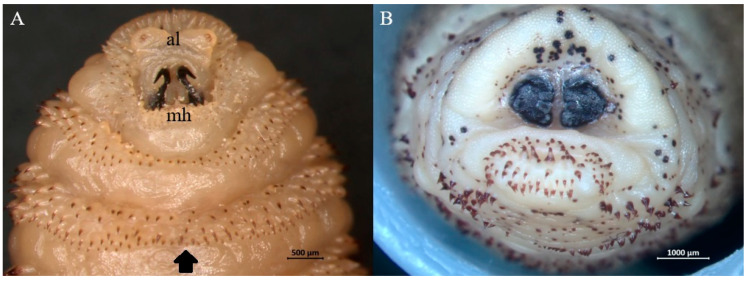
Representative third-instar larva of *Cephenemyia stimulator*. (**A**) Anterior end with V shaped antennal lobes (al), mouth hooks (mh) and arrays of cuticular spines (arrow). (**B**) Posterior end with reniform spiracular plates.

**Figure 5:**
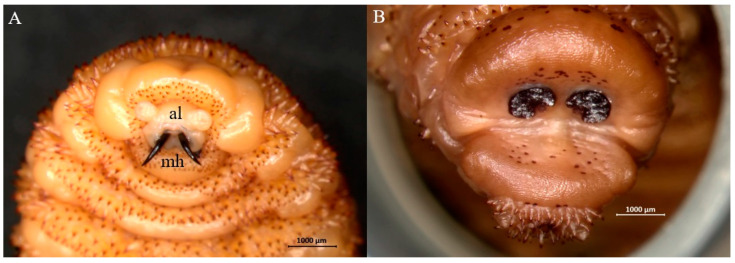
Representative third-instar larva of *Pharyngomyia picta*. (**A**) Anterior end with mouth hooks (mh) and widely separated antennal lobes (al). (**B**) Posterior end with crescent-shaped spiracular plates.

Morphological examination of third-instar larvae suggested that all belonged to one of the following three species: *H. diana* (Figure 3), *C. stimulator* (Figure 4), and *P. picta* (Figure 5). All subcutaneous larvae were identified as *H. diana*, while nasopharyngeal larvae were identified as *C. stimulator* in 27 roe deer, or *P. picta* in the one red deer. 

Taxonomic classification was further explored through the partial sequencing of the gene-encoding cytochrome oxidase subunit I. A total of 23 second-instar and 40 third-instar larvae that had been assigned to *H. diana* based on morphology were sequenced. All 63 amplicons shared the same sequence, which was >90% similar to the corresponding sequences for the following species: *H. lineatum* (GenBank accession NC013932), 90.44% similar; *H. bovis* (NC080982), 90.41% similar; *H. sinense* (NC071819), 90.29% similar; and *H. sinense* (AF295558), 90.36% similar. Similarity to previously published *H. diana* sequences could not be determined because the deposited sequences from that species came from regions of the cytochrome oxidase subunit I gene that did not overlap with the sequence that we amplified. A total of 19 second-instar and 47 third-instar larvae that had been assigned to *C. stimulator* based on morphology were sequenced. All 66 sequences were identical and 99.85% similar to the corresponding sequence reported for *C. stimulator* collected from roe deer in Spain (NC_059850). Two second-instar and two third-instar larvae of putative *P. picta* were sequenced. All sequences were the same and 99.70% similar to *P. picta* obtained from red deer in Austria (KX146940). 

**Figure 6:**
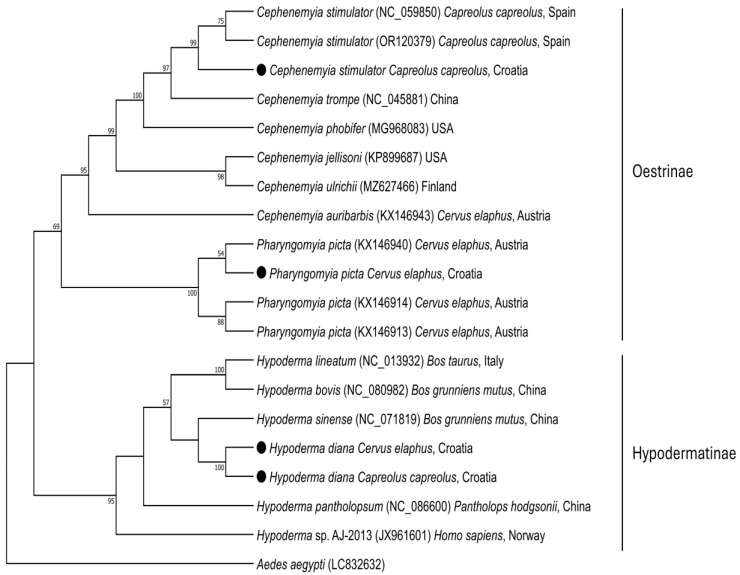
Maximum-likelihood phylogenetic tree based on cox1 nucleotide sequences of species in subfamilies Osterinae and Hypodermatinae. The strains identified in the present study are indicated with a solid circle. Previously reported strains of *H. diana*, *H. tarandi* and *H. actaeon* were not included because the corresponding sequences in the database come from different regions of the cox1 gene than the region sequenced in the present study.

Phylogenetics were clearly differentiated among the three botfly species (Figure 6), validating our species assignments based on the region of the cytochrome oxidase subunit I gene that we amplified.

## 4. Discussion

The obligatory myiasis of wild cervids is found in diverse regions across the globe, including southeastern Europe [1]. Despite their growing significance and expansion due to environmental changes, their distribution and prevalence in southeastern Europe is virtually unknown [8]. Our analysis of wild deer from across Croatia indicates that at least three species of myiasis-causing flies are prevalent: *H. diana*, *C. stimulator* and *P. picta*. Our results are the first attempt at detecting botflies based on the combination of morphology and genetic sequencing, which is important in light of the morphological similarities among dipteran species [22]. Our phylogenetic analysis supports the morphology-based taxonomic classification of *C. stimulator* and *P. picta* in our samples, but it did not confirm our assignment of *H. diana* because of the lack of reference sequences in GenBank.

In 1959, *C. stimulator* and *H. bovis* were reported in the nasopharynx and subcutaneous tissue of roe deer in Croatia, although those taxonomic assignments were based purely on morphology [23]. We recently detected *O. ovis* in a human and confirmed the taxonomy through sequencing [24]. The present study substantially extends our understanding of botfly species in the country. It also provides data for analyzing the prevalence of these species in other parts of southeastern Europe.

*H. diana* in our study was detected in roe deer and red deer, and all 63 samples from 17 animals collected from around the country during a 10-year period showed the same sequence in the region of the cytochrome oxidase subunit I gene that we amplified. These observations suggest that *H. diana* has not diverged into strains preferring one host or another. Such a lack of host specificity is consistent with previous studies of its prevalence in red deer (Spain) and roe deer (Romania) [25,26] or its detection in horses and alpacas in Germany [27,28]. It seems likely that *H. diana* may be present in other wild ruminants in Croatia and elsewhere in southeastern Europe. We also suggest that the *H. bovis* identified in Croatian roe deer in 1959 [23] may actually have been *H. diana*, given that no cases of deer infection with *H. bovis* have been reported since then. In the present study, we did not identify *H. actaeon* in subcutaneous tissue, despite its documented presence in northern regions in Hungary [29] that are geographically distant from the Croatian border. It is noteworthy that the Hungarian study did not provide molecular confirmation of *H. actaeon*. It has predominantly been reported in wild cervids in Spain [30] and Portugal [31,32], making its absence in our findings somewhat expected.

Our detection of *C. stimulator* is consistent with the high prevalence of this nasal botfly in roe deer across Europe, especially central Europe [8,33]. It was first reported in Croatia in 1959 [23], and more recent work detected it in up to 12% of roe deer in a mountainous region of the country as well as in the flat central region near the capital [17,18]. Batinjanin (2022) [17] also detected nasopharyngeal *C. ulrichii* in two roe deer, at least based on the morphology of three third-instar larvae from Zagreb County, which we failed to detect in 125 nasopharyngeal larvae from 27 animals nearly entirely from continental and mountain regions. It seems likely that future molecular studies in Croatia and southeastern Europe will identify *C. ulrichii* and other botfly species beyond the three we detected here. 

*P. picta* was recently detected based on morphology in Croatian red deer [34], and the present study confirms its presence across the country using molecular methods. This extends the list of European countries where the botfly has been definitively detected, which already includes Poland [35], Spain [36], Hungary [37], Austria [38], and Portugal [39]. We speculate that the species is also present in other parts of southeastern Europe. 

## 5. Conclusions

The current study is, to our knowledge, the first analysis of myiasis-causing botflies in wild cervids in Croatia that combines morphological and molecular analyses. Our work establishes *H. diana*, *C. stimulator* and *P. picta* as prevalent in many parts of the country. We believe that *C. ulrichii* and likely additional botfly species are present as well, which should be examined in future work. Such work may also uncover multiple haplotypes of these species. Our results provide a reference for similar investigations elsewhere in southeastern Europe, which are important for obtaining a complete understanding of botfly distribution throughout Europe as climate conditions become increasingly favorable to their growth. 

## Data Availability

The datasets from the current study are available upon request to the corresponding author.

## Supplementary Materials

The following supporting information can be downloaded at: https://www.mdpi.com/article/10.3390/ani15020208/s1, Table S1: Data summary from the research.
